# Sequence analysis of the 3’-untranslated region of *HSP70* (type I) genes in the genus *Leishmania*: its usefulness as a molecular marker for species identification

**DOI:** 10.1186/1756-3305-5-87

**Published:** 2012-04-28

**Authors:** Jose M Requena, Carmen Chicharro, Lineth García, Rudy Parrado, Concepción J Puerta, Carmen Cañavate

**Affiliations:** 1 Centro de Biología Molecular “Severo Ochoa” (CSIC-UAM), Universidad Autonoma de Madrid, 28049, Madrid, Spain; 2 WHO Collaborating Centre for Leishmaniasis, Servicio de Parasitología, Centro Nacional de Microbiología, Instituto de Salud Carlos III, 28220, Madrid, Spain; 3 Instituto de Investigaciones Médicas, Fac. Medicina, Universidad Mayor de San Simón (UMSS), Cochabamba, Bolivia; 4 Laboratorio de Parasitología Molecular, Facultad de Ciencias, Pontificia Universidad Javeriana, Bogotá, Colombia

**Keywords:** *Leishmania*, HSP70, 3’UTR, Sequence analysis, Microsatellites, Phylogenetic analysis

## Abstract

**Background:**

The Leishmaniases are a group of clinically diverse diseases caused by parasites of the genus *Leishmania*. To distinguish between species is crucial for correct diagnosis and prognosis as well as for treatment decisions. Recently, sequencing of the HSP70 coding region has been applied in phylogenetic studies and for identifying of *Leishmania* species with excellent results.

**Methods:**

In the present study, we analyzed the 3’-untranslated region (UTR) of *Leishmania HSP70*-type I gene from 24 strains representing eleven *Leishmania* species in the belief that this non-coding region would have a better discriminatory capacity for species typing than coding regions.

**Results:**

It was observed that there was a remarkable degree of sequence conservation in this region, even between species of the subgenus *Leishmania* and *Viannia*. In addition, the presence of many microsatellites was a common feature of the 3´-UTR of *HSP70-I* genes in the *Leishmania* genus. Finally, we constructed dendrograms based on global sequence alignments of the analyzed *Leishmania* species and strains, the results indicated that this particular region of *HSP70* genes might be useful for species (or species complex) typing, improving for particular species the discrimination capacity of phylogenetic trees based on HSP70 coding sequences. Given the large size variation of the analyzed region between the *Leishmania* and *Viannia* subgenera, direct visualization of the PCR amplification product would allow discrimination between subgenera, and a *Hae*III-PCR-RFLP analysis might be used for differentiating some species within each subgenera.

**Conclusions:**

Sequence and phylogenetic analyses indicated that this region, which is readily amplified using a single pair of primers from both Old and New World *Leishmania* species, might be useful as a molecular marker for species discrimination.

## Background

Protozoan parasites of *Leishmania* genus cause a severe disease, Leishmaniasis, which threatens 350 million people worldwide and some 2 million new cases occur yearly. Furthermore, mortality and morbidity from Leishmaniasis worldwide show a worrying increasing trend [1]. Infection by the parasite *Leishmania* can result in a broad spectrum of pathological outcomes in the human host, ranging from simple self-healing cutaneous lesions (cutaneous Leishmaniasis, CL) to acute visceral Leishmaniasis (VL), commonly referred to as kala-azar. The different pathologies usually correlate with infection by different species [2]. Thus, *Leishmania donovani* and *Leishmania infantum* usually cause VL, whereas the rest of *Leishmania* species generally cause cutaneous Leishmaniasis (CL). Nevertheless, exceptional cases have been described, such as visceral outcomes in individuals infected with *L. tropica*. After self-cure or successful chemotherapy, many patients remain asymptomatically infected; however, such persistence often gives rise to disease reactivation, including post-kala-azar dermal Leishmaniasis after cure of VL caused by *L. donovani*, diffuse cutaneous Leishmaniasis (DCL) lesions in *L. mexicana*-infected people, or the development of destructive mucocutaneous Leishmaniasis (espundia) months or years after healing of a localized cutaneous ulcer caused by *L. braziliensis*[1].

On the other hand, treatment outcome in patients can vary with the infecting *Leishmania* species because of different sensitivity of the parasite species both to the standard drugs, pentavalent antimonials and miltefosine, and those on clinical trial, paromomycin [3]. Thus, correct diagnosis of the infecting *Leishmania* species is crucial for making decisions regarding prognosis and treatments, and also for epidemiological monitoring of the parasite spread [4].

To date, around 20 *Leishmania* species are known to infect humans, even though the species status is under discussion for some of them [1,5]. In fact, a major conceptual problem in epidemiology of Leishmaniasis is the taxonomy of the etiological agent as *Leishmania* is a morphologically uniform genus. At present, multilocus enzyme electrophoresis (MLEE), an isoenzyme analysis that is based on 13 enzymes, is the reference method for *Leishmania* typing [1]. However, MLEE has important limitations: strains with the same enzyme phenotype may have distinct amino acid sequences, the degree of relationship between different phenotypes is not known, and putative heterozygous phenotypes are difficult to interpret [6,7]. Furthermore, this technique is time consuming, requires mass parasite culturing, and it should be done by one of the few reference centres [1]. Therefore, its usefulness for taxonomic purposes is under dispute [8]. In conclusion, new methods for species identification are needed to ensure proper identification and therapy.

Molecular biology techniques, such as PCR and DNA sequencing, are replacing traditional methods of taxonomy, as they tend to be more specific and easier to perform. Thus, sequencing of genes such as DNA polymerase α [9], RNA polymerase II [10], gp63 [11], cytochrome oxidase II [12,13], N-acetylglucosamine-1-phosphate transferase [14], cystein protease b [15], cytochrome b [16,17], SSU rDNA [18], and 7SL RNA [19] has been carried out for phylogenetic studies. In addition, sequencing of ribosomal DNA internal transcribed spacers [20,21] and kinetoplast DNA minicircles [22] is being used for taxonomic purposes within the *Leishmania* genus. Remarkably, in recent studies, coding regions of genes for most metabolic enzymes used in the MLEE typing method have been determined and used for *Leishmania* typing [6,23,24]. As expected, these studies have demonstrated that DNA sequencing has greater discriminatory power than MLEE typing. Accordingly, this approach, based on sequence analysis of selected genes, has been dubbed MLST (for “multilocus sequence typing”) [6].

The HSP70 gene is being extensively used as a target for PCR-RFLP assays for *Leishmania* species discrimination [25,26]. In fact, Montalvo and co-workers [27] have designed a PCR-RFLP method allowing identification of most medically relevant Old and New World *Leishmania* species on the basis of *HSP70* PCR amplification. More recently, sequence analysis of the 1380-bp fragment of the *HSP70* coding region, commonly used in the PCR-RFLP method, has been directly used for phylogenetic and taxonomic purposes [26,28]. The phylogenetic trees obtained by da Silva *et al.*[26] and Fraga *et al.*[28] demonstrate that DNA sequencing of HSP70 coding regions represent a valuable technique for *Leishmania* species identification. As a remarkable result from these studies, Fraga and coworkers [28] defined eight, highly supported, monophyletic groups within the genus *Leishmania*: (1) *L. donovani/L. infantum*, (2) *L. tropica/L. aethiopica*, (3) *L. major*, (4) *L. mexicana/L. amazonensis*, (5) *L. naiffi*, (6) *L. braziliensis/L. peruviana*, (7) *L. guyanensis/L. panamensis*, and (8) *L. lainsoni*. Nevertheless, the HSP70 protein and the coding region of its gene are among the best conserved sequences along the evolutionary tree of life [29]. As untranslated regions (UTR) of genes are expected to have a lower evolutionary constriction than coding regions (CDR), it is conceivable that UTR sequences may be a better target than CDR for typing of closely related organisms [30]. In the present study, we sequenced most of the 3’-untranslated region (UTR) of *Leishmania HSP70*-type I (*HSP70-I*) gene from different species and strains with the belief that this region might be useful for species typing within the genus, having hopefully a more discriminating capacity than the HSP70 coding region.

## Methods

### DNA extraction, amplification, and cloning

**Figure 1:**
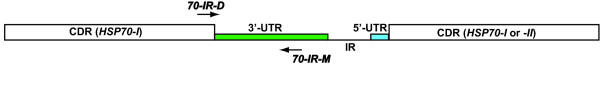
**Schematic map of the*Leishmania HSP70*locus (fragment), showing the region amplified in this study.** The map was drawn to scale [39]. PCR primer annealing sites are indicated by arrows. CDR, protein coding region; 5’- and 3’-UTR, untranslated regions; IR, intergenic region.

The *Leishmania* isolates used in this study are listed in Table 1. Promastigotes were cultivated in RPMI medium supplemented with 10% heat-inactivated fetal bovine serum at 26°C. Total genomic DNA was extracted from promastigotes using a standard sodium dodecyl sulfate-proteinase K procedure as previously described [31]. For amplification, the primers 70-IR-D (5’- CCAAGGTCGA GGAGGTCGAC TA- 3’) and 70-IR-M (5’- ACGGGTAGGG GGAGGAAAGA -3’) were used (Figure 1). Amplifications were done with the Maxime PCR PreMix Kit (i-Taq; Intron Biotechnology), using 20–40 ng of genomic DNA, dNTPs (2.5 mM each) and 0.5 μM of each primer. The PCR profile was 95°C for 2 min followed by 30 cycles of 95°C for 30 sec, 62.5°C for 30 sec, 72°C for 1 min and 20 sec, and then a final elongation step at 72°C for 5 min. The PCR products were checked by agarose gel electrophoresis and purified using a commercial DNA purification kit (FavorPrep™ Gel/PCR Purification Kit; Favorgen), following the manufacturer’s instructions.

**Table 1:** Reference strains and sequences used in this study

**Species**	**WHO code**	**Origin**	**Sequence ID**	**Size (bp)**	**Label**^a^
*L. aethiopica*	MHOM/ET/82/101-82	Ethiopia	HE575325^b^	696^e^	Lae-101
*L. aethiopica*	MHOM/ET/82/652-82	Ethiopia	HE575326^b^	701	Lae-652
*L. aethiopica*	MHOM/ET/72/L100	Ethiopia	HE575327^b^	700	Lae-L100
*L. amazonensis*	MHOM/BR/77/LTB0016/C1S1	Brazil	L14605^c^	703^e^	Lam-LBT
*L. amazonensis*	IFLA/BR/67/PH8	Brazil	HE575328^b^	700	Lam-PH8
*L. braziliensis*	MHOM/BR/75/M2904	Brazil	Contig LbrM28_V2_October (1081948–1082464, minus strand)^d^	517^e^	Lb-M2904
*L. braziliensis*	MHOM/BO/00/CUM45	Bolivia	HE575329^b^	518	Lb-CUM45
*L. donovani*	MHOM/IN/80/DD8	India	HE575330^b^	718	Ld-DD8
*L. donovani*	MHOM/IN/00/DEVI	India	HE575331^b^	724	Ld-DEVI
*L. donovani*	MHOM/ET/67/HU3	Ethiopia	HE575332^b^	711^e^	Ld-HU3
*L. donovani*	IMRT/KE/62/LRC-L57	Kenya	HE575333^b^	715	Ld-LCR
*L. guyanensis*	MHOM/PE/91/LC1446	Peru	HE575334^b^	585^e^	Lg-LC1446
*L. infantum*	MCAN/ES/98/LLM-877 (JPCM5)	Spain	Contig LinJ28_20070420_V3 (1094043–1094771, minus strand)^d^	729^e^	Li-JPCM5
*L. infantum*	MHOM/FR/78/LEM-75	France	HE575335^b^	731	Li-LEM75
*L. infantum*	MCAN/ES/01/LLM-1050	Spain	HE575336^b^	733	Li-LLM1050
*L. infantum*	MHOM/ES/05/LLM-1525	Spain	HE575337^b^	731	Li-LLM1525
*L. lainsoni*	MHOM/BO/95/CUM71	Bolivia	HE575338^b^	589^e^	Ll-CUM71
*L. major*	MHOM/IL/80/Friedlin	Israel	LmjF28_01_20050601_V5.2 (1059883–1060597, minus strand)^d^	715^e^	Lmj-Friedlin
*L. major*	MRHO/SU/59/P-STRAIN	Former Soviet Union	HE575339^b^	715	Lmj-P-STRAIN
*L. major*	MHOM/SU/73/5-ASKH	Former Soviet Union	HE575340^b^	718	Lmj-5-ASKH
*L. panamensis*	MHOM/COL/81/L13	Colombia	LpnLscaffold351 (149539–150125, minus strand)^f^	587	Lpa-L13
*L. peruviana*	MHOM/PE/90/LH249	Peru	HE575341^b^	516^e^	Lp-LH249
*L. tropica*	MRAT/IQ/72/ADHANIS1	Iraq	HE575342^b^	704^e^	Lt-ADHANIS1
*L. tropica*	MHOM/SU/74/K27	Former Soviet Union	HE575343^b^	702	Lt-K27

^a^Label for strain identification in Figure 3.

^b^New sequences submitted to EMBL-EBI database.

^c^GenBank accession number.

^d^GeneDB identificator.

^e^Sequences depicted in Figure 2.

^f^TriTrypDB identificator (http://www.tritrypdb.org).

**Figure 2:**
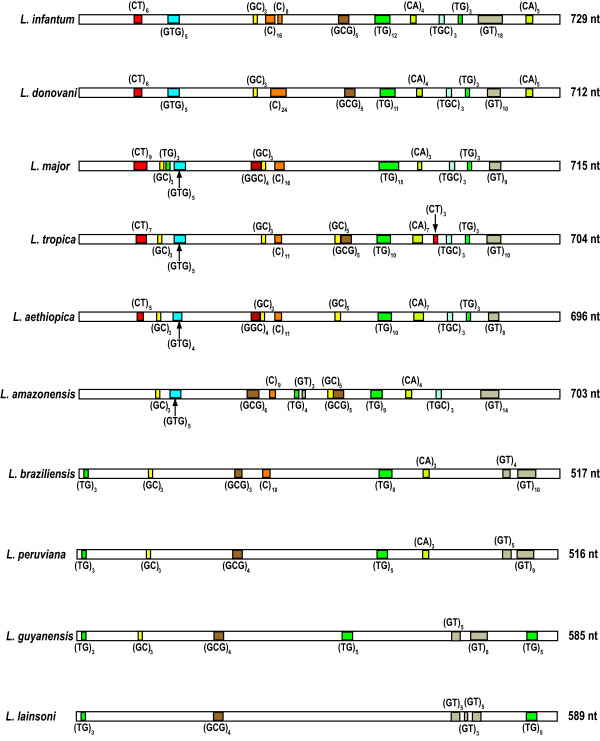
**Microsatellite distribution in the 3’-UTR of*HSP70-I*genes in several*Leishmania*species.** The repeated motifs correspond to those found in the sense strand. Note that the drawing scale is not proportional for the different *Leishmania* species; however, for a given sequence, the relative positions of the microsatellites are scaled.

For cloning, the purified PCR products were ligated into the pCR2.1 vector using the TA Cloning Kit (Invitrogen), and the ligation product used to transform XL1-blue competent bacteria [35]. DNA sequencing was carried out in the facilities of the Servicio de Genómica (Parque Tecnológico de Madrid-UAM), using Big Dye Terminators v3.1 kit (Applied Biosystem).

### Sequence analysis

The chromatograms were visualized using the BioEdit Sequence Alignment Editor [36]. For all the clones, sequencing of both strands was carried out.

For all analyses, the priming sites were trimmed from both ends of all sequences. A search for microsatellite sequences was performed using the “Microsatellite repeats finder” program [37]. Parameter settings were: length of repeated sequence (minimum, 2; maximum, 6); minimum number of repeats, 3; minimum length of tandem repeat, 6; and allowed percentage of mismatches, 10%.

All sequences were multiple aligned with the default option using ClustalW2.1 at The European Bioinformatics Institute [33]. For phylogenetic studies, the multiple sequence alignment was analyzed using the software MEGA4 [32]. The evolutionary history was inferred using the Neighbor-Joining and Minimum Evolution methods [34], and the bootstrap consensus tree inferred from 1000 replicates. The evolutionary distances were computed using the Maximum Composite Likelihood method [38] and the units represent the number of base substitutions per site. All positions containing gaps and missing data were eliminated from the dataset.

### Sequence data

Sequences generated in this study have been deposited in the EMBL-EBI database under accession numbers HE575325-HE575343 (Table 1).

## Results and discussion

### PCR amplification of non-coding sequences of *Leishmania HSP70-I* genes

The *HSP70* locus in *Leishmania* consists of several *HSP70* gene copies arranged in a head-to-tail manner [39,40]. All the genes are highly conserved in sequence with the exception of the gene located at the 3’ end of the cluster, which presents a 3’-UTR different to the rest of genes. Hence, the latter gene is referred to as *HSP70-II* gene and the other genes as *HSP70-I* genes. Given the high sequence conservation of *HSP70* CDR among the different *Leishmania* species [41], we designed oligonucleotides at the 5’ and 3’ extremities of the CDR with the purpose of PCR amplify the entire intercoding region of *HSP70-I* genes. The intercoding region (3’-UTR + intergenic region (IR) + 5’-UTR) in the *L. infantum HSP70* locus is 1600 bp in length [39]. However, the task turned out to be complicated, as it was only possible to amplify and clone the expected region for a few *Leishmania* species. In order to overcome this difficulty, we searched for conserved sequence blocks within the 3’-UTR of *HSP70-I* genes from different *Leishmania* species, whose sequences were available in the databases. Finally, we succeeded and designed an oligonucleotide pair able to efficiently amplify a 3’-UTR sequence from all the *Leishmania* DNA samples, irrespective of the subgenus (*Leishmania* or *Viannia*) to which they belonged. See Figure 1 for details on the location of amplification primers 70-IR-D and 70-IR-M in the *HSP70* locus.

The UTR-I fragment for 20 strains of different *Leishmania* species were PCR-amplified and cloned. However, it was not possible to obtain a complete sequence for the strain MHOM/GR/80/GR-L35 of *L. tropica*, even after using alternative sequencing protocols in which DMSO or betaine was added [42]. As a result, this strain was not included in subsequent studies. For many of the other sequences, the sequence chromatogram was not uniform, showing a drastic decrease in intensity after passing GC-rich regions. This fact may be an indication that the UTR-I fragment contains regions with strong secondary structures that were hindering (or totally blocking in one case, as mentioned above) the polymerase advance during sequencing reaction.

In addition to the 19 sequences obtained in this work, we retrieved 5 sequences from databases, amounting to a total of 24 sequences (Table 1). It should be noted that sequence information of this region is not yet available in *L. mexicana*[43]*, L. donovani*[43], and *L. tarentolae*[44] genome sequencing projects, which are ongoing. The *HSP70-UTR-I* amplified region varied in size from 516 (in *L. peruviana*) to 733 bp (in *L. infantum*). Thus, the region is clearly shorter in *Leishmania* species of the subgenus *Viannia* than in species of the subgenus *Leishmania*, and this fact might be exploited as a PCR-based method for quick discrimination between both *Leishmania* subgenera. Within a given species, the length of this region shows a very low inter-strain variation; for example, the size of the region in four different *L. donovani* strains varies from 711 to 718, in *L. infantum* (four strains) ranges from 729 to 733, in *L. major* (3 strains) ranges from 715 to 718, and in *L. aethiopica* (3 strains) ranges from 696 to 701. Within a given *Leishmania* species, size variants are a consequence of variations in the repeat number of microsatellite sequences (see Figure 2).

### The *HSP70 UTR-I* contains a remarkable abundance of microsatellites

A search for structural motifs along the sequenced UTR-I fragment in the different *Leishmania* species was carried out. The study showed a significant richness in microsatellites sequences (Figure 2). Microsatellites are tandemly repeated stretches of short nucleotide motives of 1–8 bp ubiquitously distributed in eukaryotic genomes [45]. The *Leishmania* genome is not an exception, and multilocus microsatellite typing (MLMT) is being used as a powerful tool for population genetic and epidemiological studies of *Leishmania*[46]*.* In the *L. infantum, L. major* and *L. braziliensis* genomes, the CA repeats represent by far the most common microsatellite [47]. Indeed, CA-microsatellites (read as TG or GT on the complementary strand) were the more abundant class in the UTR-I of all *Leishmania* species, showing most of them have an antisense orientation (Figure 2). TGC-microsatellites, another abundant repeat in *Leishmania* genomes [47], were found in the *Leishmania* species of the subgenus *Leishmania* but absent in the species of the subgenus *Viannia*. However, the analyzed *HSP70 UTR-I* fragment contains many other microsatellites (C, GC, GTG, GGC, GCG) that may be categorized as uncommon for *Leishmania*.

**Figure 3:**
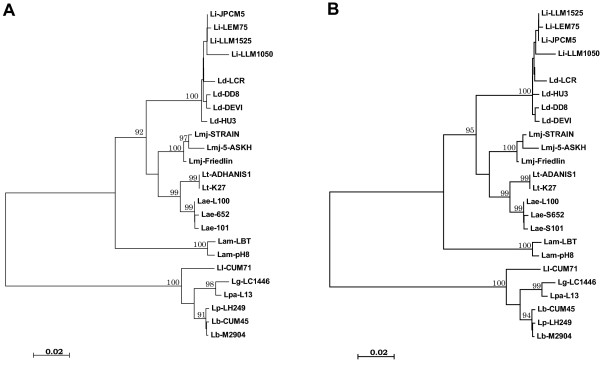
**Phylogeny of*Leishmania*according to the Neighbor-joining (panel A) and Minimum Evolution (panel B) methods based on the multiple sequence alignment of 3’UTR of*HSP70-I*gene.** Numbers above the branch represent percent recovery in 1,000 bootstrap pseudoreplicates analysis (only values above 90 are shown). All positions containing gaps and missing data were eliminated from the dataset (complete deletion option). There were a total of 493 positions in the final dataset. Phylogenetic analyses were conducted in MEGA4 [32]. Distance represents the number of base substitutions per site. Species are abbreviated as follows: Lae, *L. aethiopica*; Lam, *L. amazonensis*; Lb, *L. braziliensis*; Ld, *L. donovani*; Lg, *L. guyanensis*; Li, *L. infantum*; Ll, *L. lainsoni*; Lmj, *L. major*; Lp, *L. peruviana*; Lpa, *L. panamensis*; Lt, *L. tropica*. See Table 1 for additional information of the strains.

The HSP70 locus in *T. cruzi, T. brucei* and *Leishmania* spp. has a similar genomic organization, i.e. it contains several genes that are head-to-tail tandemly organized. However, the UTRs of *HSP70* genes are extremely divergent between *Trypanosoma* and *Leishmania* genera. Thus, the 3’UTR of HSP70 genes in *T. brucei* and *T. cruzi* are only 186 and 213 nucleotides in length, respectively [48,49]. In contrast, the 3’-UTR of *Leishmania* HSP70 genes are higher than 1000 nucleotides in length for species of the *Leishmania* subgenus [39], and 936 nucleotides for the 3’-UTR-I in *Leishmania braziliensis*[30]. These findings suggest that UTRs of HSP70 genes have evolved towards opposite ends regarding sequence complexity in *Trypanosoma* and *Leishmania*. On the other hand, from the analysis of Figure 2, it became clear that the analyzed region of the *HSP70* 3’UTR has experienced also a notable evolution after separation of subgenus *Leishmania* and *Viannia*. In the species of the subgenus *Leishmania*, this region has grown both in size and microsatellite complexity. Interestingly, the regulation of *HSP70-I* expression seems to be more complex in species of the subgenus *Leishmania* than in *L. braziliensis*[40]. Thus, it would be interesting to analyze the contribution of the different microsatellites to the specific regulation of *HSP70-I* genes in *Leishmania*.

### Phylogenetic relationships of *Leishmania* species based on sequences of the *HSP70-I* UTR

From the structural analysis of this region in the different *Leishmania* species, it became evident that this fragment of *HSP70-I* genes has greatly diverged between the subgenus *Leishmania* and *Viannia* (Figure 2). In order to study the usefulness of these sequences to discriminate at the level of species within each subgenus, phylogenetic analyses were carried out (Figure 3). The inferred phylogenetic tree based on either the Neighbor-Joining or Minimum Evolution methods indicated that the sequenced region may be useful for clustering *Leishmania* strains at the level of species or complex of species (99 to 100% bootstrap support). Not surprisingly, the subgenera *Leishmania* and *Viannia* constitute two distinct monophyletic lines. In addition, our phylogenetic analyses gave a robust support for the following *Leishmania* species or clusters: *L. amazonensis* (which forms a separated branch from the Old World *Leishmania* species), *L. infantum/L. donovani (L. donovani* complex), *L. majorL. tropica* and *L. aethiopica*, within the *Leishmania* subgenus; and *L. braziliensis/L. peruviana* (*L. braziliensis* complex), *L. guyanensis/L. panamensis* (*L. guyanensis* complex), and *L. lainsoni*, within the *Viannia* subgenus. With few differences, our phylogenetic tree was coincident with that obtained by Fraga *et al.*[28] using sequences of the *Leishmania HSP70* CDR. Nevertheless, our phylogenetic results support a separation between *L. tropica* and *L. aethiopica* (99% of bootstrap value; Figure 3), whereas they cannot be distinguished based on *HSP70* coding sequences.

Separation between *L. donovani* and *L. infantum* strains remains as an issue to be resolved, even though several comprehensive studies have been conducted [20,46,50-53]. In our phylogenetic tree (Figure 3), separation of *L. donovani* and *L. infantum* strains was observed, even though the bootstrap value (50-52%, depending on the phylogenetic method) was not statistically significant. In the future, we plan to analyze a large number of *L. donovani* and *L. infantum* strains in order to define signatures within the 3’-UTRI that could allow discriminating specifically among both species (or subspecies).

Although sequencing is becoming a cost effective methodology, it is still not feasible for routine diagnosis, being more attractive other methodologies such as restriction fragment length polymorphisms of PCR amplicons (PCR-RFLP). This methodology has been found to be valuable for *Leishmania* species discrimination, using amplicons from the *HSP70* coding region [26,27]. We analyzed the sequence of the UTR-I amplicons described in this study searching for restriction enzyme cleavage sites that could be used for PCR-RFLP-based identification of *Leishmania* species. On the basis of digestion with *Hae*III restriction enzyme, our in silico analyses predicted seven different patterns (Table 2) that would allow the separation of the following species of groups of species: *L. aethiopica /L. tropica, L. amazonensis, L. braziliensis/L. peruviana, L. donovani/L. infantum, L. guyanensis/L. panamensis, L. lainsoni and L. major.*

**Table 2:** **In silico prediction of*Hae*III restriction fragments of the*HSP70-I*UTR amplification product in the*Leishmania*species analyzed in this study**

***Leishmania*spp**	**PCR product size (pb)**	***Hae*III restriction fragments**	**Fragment sizes (pb)**
*L. aethiopica*	738	3	317, 243, 178
*L. amazonensis*	745	3	414, 221, 110
*L. braziliensis*	559	5	229, 103, 99, 81, 47
*L. donovani*	754	4	435,167,125, 27
*L. guyanensis*	627	3	442, 104, 81
*L. infantum*	771	4	436, 182, 126, 27
*L. lainsoni*	631	4	399, 104, 81, 47
*L. major*	757	4	323, 186, 178, 70
*L. panamensis*	629	3	444, 104, 81
*L. peruviana*	558	5	227, 104, 99, 81, 47
*L. tropica*	746	3	321, 243, 182

## Conclusions

Identification of the exact parasite species isolated from human and animal hosts, as well as from sand fly vectors, gains increasing importance for diagnostics, epidemiological surveillance, and clinical studies. As sequencing is becoming a cost-effective methodology, it is possible to envision the use of a battery of genes as genomic markers for phylogenetic analyses of *Leishmania* species towards the goal of both a molecular taxonomy and a definitive diagnostic method. Our data show the potential of the UTR of *HSP70-I* genes as a molecular marker for *Leishmania* species typing. In addition, this study has shown the existence of complex arrays of microsatellites in the UTR of HSP70-I, whose involvement in regulatory mechanisms of gene expression will be analyzed in future studies.

## Abbreviations

MLEE, Multilocus enzyme electrophoresis; UTR, Untranslated region; CDR, Coding region.

## Competing interests

The authors declare that non-financial competing interests exist.

## Authors' contributions

CC and JMR conceived and designed the experiments. CCh and JMR carried out the molecular genetic studies. LG, RP and CJP contributed DNA samples and analysis tools. JMR performed the sequence data analyses and drafted the manuscript. CC helped to finalize the manuscript. All authors read and approved the final manuscript.
